# Cold Atmospheric Plasma Enhances Fn14 Signaling in Hair Follicle Stem Cells, Thereby Promoting the Healing of Diabetic Skin Wounds in a Mouse Model

**DOI:** 10.1155/jimr/9082774

**Published:** 2026-05-05

**Authors:** Xiaoyan Zou, Lingling Peng, Mai Luo, Ziqing Qu, Guanglei Hu, Xiaoming Liu, Zhu Yan, Shruti Pokharel, Yumin Xia, Fangyan Jia

**Affiliations:** ^1^ Department of Dermatology, The Second Affiliated Hospital of Xi’an Jiaotong University, Xi’an, 710004, China, xjtu.edu.cn; ^2^ Department of Dermatology, Maternal and Child Health Hospital of Hubei Province, Wuhan, 430064, China; ^3^ Department of Dermatology, The First Affiliated Hospital of Xi’an Medical University, Xi’an, 710082, China, xjtu.edu.cn; ^4^ Core Research Laboratory, The Second Affiliated Hospital of Xi’an Jiaotong University, Xi’an, 710004, China, xjtu.edu.cn; ^5^ Department of Dermatology, Southern University of Science and Technology Hospital, Shenzhen, 518055, China, sustc.edu.cn

**Keywords:** cold atmospheric plasma, diabetic wound, Fn14, hair follicle stem cell, reactive oxygen species, TWEAK

## Abstract

The healing of diabetic skin wounds is often delayed due to cellular dysfunction and oxidative stress. Recently, cold atmospheric plasma (CAP) has shown promising efficacy in treating diabetic wounds; however, its exact mechanism remains unclear. The study aimed to investigate the effect of CAP on fibroblast growth factor‐inducible 14 (Fn14) signaling‐mediated hair follicle stem cell (HFSC) functionality, which may contribute to the healing of diabetic wounds. In vitro experiments were conducted to analyze the effect of CAP treatment on HFSC proliferation, cytokine production, and reactive oxygen species (ROS)‐related protein expressions. Animal experiments were performed to verify the effect of CAP treatment on a mouse type 1 diabetic wound model. CAP enhanced both Fn14 expression and HFSC proliferation, accompanied by the activation of the Wnt/β‐catenin signaling pathway. Expressions of Sirt1, Nrf2, hypoxia‐inducible factor‐1α, and Sox9, which were initially suppressed under high glucose conditions, were significantly restored upon CAP stimulation. Moreover, CAP‐activated hydrogel promoted diabetic wound healing in this mouse model. Furthermore, inhibition of Fn14 abrogated the promotive effect of CAP intervention. Fn14 signaling is a key mediator, mechanistically linking CAP to HFSC activation and tissue repair. These findings provide a therapeutic blueprint for CAP‐based regeneration of diabetic wounds.

## 1. Introduction

Diabetes mellitus is a chronic metabolic disorder associated with a variety of complications, among which cutaneous ulcers are among the most common [1]. Recent epidemiological reports indicate that the global incidence of cutaneous ulcers in patients with diabetes mellitus is ~2.2% [2]. The biology of diabetic wound healing is complex, involving impaired angiogenesis, chronic inflammation, peripheral neuropathy, bacterial infection, and metabolic dysfunctions [3]. Typically, skin wound healing—especially in acute wounds—proceeds through four sequential and coordinated phases: hemostasis, inflammation, proliferation, and remodeling [4]. However, the intrinsic biological abnormalities in diabetic wounds disrupt these physiological processes, often resulting in delayed healing, chronic disease progression, or treatment‐resistant conditions [2, 3]. Therefore, there is an urgent clinical need to identify molecular regulators involved in these pathological mechanisms and to develop innovative therapeutic strategies for effective diabetic wound management.

Tumor necrosis factor (TNF)‐like weak inducer of apoptosis (TWEAK) is a proinflammatory cytokine predominantly secreted by infiltrating macrophages [5]. As a member of the TNF superfamily, TWEAK exerts its effects by binding to its receptor, fibroblast growth factor‐inducible 14 (Fn14). TWEAK/Fn14 interaction regulates multiple cellular responses, including proliferation, angiogenesis, and inflammatory cytokine induction [5, 6]. Under normal physiological conditions, tissue‐resident cells express minimal levels of Fn14. However, this transmembrane receptor is significantly upregulated in inflamed tissue microenvironments and is constitutively expressed at high levels in various stem cell populations [6]. Fn14 expression has been observed in keratinocytes during autoimmune inflammation and in dermal microvascular endothelial cells in psoriatic lesions [7, 8]. Additionally, TWEAK/Fn14 signaling plays a role in burn wound healing by regulating the functions of dermal fibroblasts [9]. Moreover, TWEAK not only maintains basal functions but also activates the regenerative capacity of various stem and progenitor cells, which exhibit persistent Fn14 expression even in the absence of inflammatory stimuli [5, 6]. Several types of stem cells contribute to skin wound healing, including ductal progenitor cells, epidermal stem cells, and hair follicle stem cells (HFSCs) [4]. TWEAK facilitates the migration of endothelial progenitor cells to wound sites and enhances healing by promoting their endothelial maturation [6, 10]. In skin injuries, macrophages and neutrophils release TWEAK, which binds to Fn14 and modulates the activity of interfollicular epidermal stem cells [11]. These findings underscore the critical role of TWEAK/Fn14 signaling in regulating cutaneous stem cell functions and promoting skin wound healing.

Despite advancements in wound care, certain skin wounds—such as diabetic ulcers—remain refractory to current therapies. Wound management is as crucial as treatment in clinical practice, highlighting the need for novel therapeutic approaches to improve outcomes. Recently, cold atmospheric plasma (CAP) therapy has been applied in patients with diabetic foot ulcers, demonstrating beneficial effects in randomized clinical trials [12, 13]. CAP is a form of plasma (the fourth state of matter) that contains both positively and negatively charged species. It operates at atmospheric pressure and near‐room temperature. CAP may improve skin conditions by modulating reactive oxygen species (ROS)–mediated processes [14]. Animal studies have revealed that CAP exerts therapeutic effects through various mechanisms, including inflammation modulation, antioxidant activity, tissue remodeling, and antimicrobial activity [12, 13, 15]. However, research on CAP therapy for diabetic skin wounds remains limited, particularly regarding its effects on key proinflammatory mediators and stem cell functionality. Given the established involvement of TWEAK and HFSCs in skin wound healing, this study tests the hypothesis that CAP promotes diabetic wound healing by enhancing Fn14‐mediated signaling in HFSCs, thereby restoring their regenerative capacity. On the other hand, this study aimed to investigate the regulatory effects of CAP on Fn14 signaling and HFSC function in the context of diabetic wound healing.

## 2. Material and Methods

### 2.1. Ethical Approval

This study was conducted with the ethical approval (No.2021‐1019) from the Ethical Committee of Xi’an Jiaotong University Medical School. The protocols used strictly followed the Animal Research: Reporting of In Vivo Experiments guidelines.

### 2.2. Cell Culture, Transfection, and Proliferation Analysis

Human primary HFSCs were isolated from scalp specimens following established protocols [16, 17]. The isolation involved a two‐step enzymatic digestion followed by differential centrifugation for cell enrichment. Specifically, the follicular bulge region, located between the isthmus and upper hair bulb, was carefully dissected using microsurgical scissors under a stereomicroscope (Leica, Wetzlar, Germany). Isolated cells were identified as HFSCs based on immunofluorescence detection of integrin β1 and K15 expressions [17], and cultured in keratinocyte serum‐free medium (Thermo Fisher Scientific, Waltham, MA, USA) supplemented with Y‐27632 (MedChemExpress LLC, Princeton, NJ, USA).

For small interfering RNA (siRNA) transfection, HFSCs were transfected with either control siRNA or *Fn14*‐targeting siRNA (Life Technologies, Grand Island, NY, USA) using Lipofectamine 2000 at a ratio of 15 pmol RNA to 1.5 μL reagent. Transfection efficiency was confirmed by both Western blotting and immunofluorescence (Figure S1).

Some cells were cultured in the above medium supplemented with high glucose (33.3 mmol/L) to recapitulate the diabetic microenvironment, including oxidative stress or inflammatory responses [18]. Cell proliferation was assessed using the Cell Counting Kit‐8 (CCK‐8; Beyotime Company, Shanghai, China). Briefly, cells (1 × 10^5^ cells/mL) were treated with 10 µL of CCK‐8 reagent. After 2‐h incubation, optical density was measured at 450 nm using a microplate reader.

### 2.3. Mouse Model of Diabetic Wounds

Fn14 deficiency in Balb/c mice was achieved via the clustered, regularly interspaced, short palindromic repeats (CRISPR)/CRISPR‐associated (Cas) 9 genome editing [9]. A type 1 diabetic wound model was established as previously described [19]. Male Balb/c mice (10 weeks old) received intraperitoneal injections of 50 mg/kg streptozotocin (Psaitong, Beijing, China) in 0.1 M sodium citrate buffer for five consecutive days. Two weeks post‐injection, mice with blood glucose >16.7 mM were considered to indicate diabetic status [19]. After a 4‐week stabilization period, mice were anesthetized by intraperitoneal injection of ketamine (90 mg/kg) and xylazine (10 mg/kg), followed by creation of full‐thickness dorsal wounds (10 mm in diameter). Wound healing progression was photographed, and relative wound area (%) was calculated using ImageJ as (Area_n/Area_0) × 100, where Area_0 and Area_n represent the wound area at day 0 and subsequent days (3, 7, 14).

### 2.4. CAP Treatment

The CAP device used in this study was a surface discharge plasma generator (Shaanxi SXY Medical Electronics Company, China). Its composition and technical parameters were detailed elsewhere [20]. For in vitro studies, culture media were pretreated with CAP for 0–30 min and then immediately applied to cells for 24‐h incubation (Figure S2). These concentrations of H_2_O_2_, NO, NO_2_^–^, and NO_3_^–^ were determined in culture media by using the H_2_O_2_ or nitrate/nitrite assay kits (Beyotime; Figure S3). In another setup, the cells were cultured in CAP‐activated media (using the same method for a fixed 30‐minute pretreatment) and subsequently incubated for up to 48 h.

For in vivo treatment, diabetic mice received a CAP‐activated hydrogel (Figure S2). The hydrogel comprised a polyacrylamide‐based copolymer, synthesized with taurine and polyvinylpyrrolidone (Tomer Cosmetic Co., Guangzhou, China). Before being topically applied to wounds, the gel was positioned 1 cm from the CAP discharge interface and exposed for 10 min. The 10‐min CAP treatment duration was determined based on our previous findings [14, 21], which demonstrated that a treatment period of 5–20 min achieves an optimal balance between short‐lived and long‐lived reactive species generated in the gel during CAP exposure. Treatment was administered once daily for 2 weeks. Wound areas were monitored, and tissues were collected at the endpoint. Euthanasia was performed by intraperitoneal injection of sodium pentobarbital (150 mg/kg).

### 2.5. Quantitative Real‐Time Polymerase Chain Reaction (qRT‐PCR)

Total RNA was extracted from cell cultures immediately after termination of stimulation or treatment using TRIzol reagent (Invitrogen, Carlsbad, CA, USA). Complementary DNA (cDNA) was synthesized using a Takara Bio kit (Kyoto, Japan), followed by qRT‐PCR with SYBR Green Master Mix (Takara Bio) on an ABI PRISM 7900HT system (Applied Biosystems, Waltham, MA, USA). All primers (Sangon Biotech, Shanghai, China) are listed in Table S1. Gene expression was quantified using the 2^–△△Ct^ method.

### 2.6. Immunofluorescent Assay

HFSCs grown in glass‐bottom dishes (MatTek, Ashland, MA, USA) were fixed with 4% paraformaldehyde, permeabilized with 0.2% Triton X‐100, and blocked with 2% goat serum. Cells were incubated overnight at 4°C with rabbit Alexa Fluor 488‐conjugated antibody targeting β‐catenin, Ki67 or Sox9, followed by nuclear staining with 4^′^, 6‐diamidino‐2‐phenylindole (DAPI; Beyotime). For immunofluorescence detection of Fn14 expression, the primary and secondary antibodies are mouse anti‐Fn14 IgG and Alexa Fluor 488‐conjugated rabbit anti‐mouse IgG monoclonal antibody, respectively. Imaging was performed using a TCS SP2 confocal microscope (Leica, Wetzlar, Germany). Antibody details are listed in Table S2.

### 2.7. Flow Cytometry

Apoptosis was assessed using the propidium iodide (PI)/Alexa 488‐Annexin V Kit (Thermo Fisher Scientific). Cultured cells were trypsinized, resuspended in binding buffer, and stained for 20 min. Flow cytometry was performed using a FACSAria II with FACSDiva 7.0 software (BD Biosciences, San Diego, CA, USA). For stem cell analysis, parallel samples were stained with Alexa Fluor 488‐anti‐Sox9 antibody following the same protocol.

### 2.8. Western Blotting

Protein lysates were extracted using radio‐immunoprecipitation assay lysis buffer with protease inhibitors (Thermo Fisher Scientific). Nuclear/cytoplasmic fractions were isolated using a NE‐PER Kit (Thermo Fisher Scientific). Proteins were separated by sodium dodecyl sulfate–polyacrylamide gel electrophoresis, transferred to polyvinylidene difluoride membranes (Millipore, Billerica, MA, USA), and probed with rabbit primary antibody targeting Fn14, β‐catenin, inducible nitric oxide synthase (iNOS), sirtuin 1 (Sirt1), nuclear factor E2‐related factor 2 (Nrf2), hypoxia‐inducible factor (HIF)‐1α, Sox9 or β‐actin (2 µg/mL) followed by horseradish peroxidase‐conjugated goat anti‐rabbit IgG (2 µg/mL; Abcam). Detection used an electrochemiluminescence kit (Millipore), with antibodies and manufacturers listed in Table S2. Band quantification was performed using ImageJ v1.54 software (National Institutes of Health, Bethesda, MD, USA).

### 2.9. Enzyme‐Linked Immunosorbent Assay (ELISA)

Cytokine concentrations in cell culture supernatants were quantified using ELISA kits (Abcam) for epidermal growth factor (EGF; #ab217772), transforming growth factor β1 (TGF‐β1; #ab100647), and vascular endothelial growth factor (VEGF; #ab222510).

### 2.10. Immunohistochemistry

Skin tissue was routinely processed into paraffin sections, pretreated with 2% goat serum (Beyotime), and subjected to antigen retrieval in sodium citrate buffer. Primary antibodies, including rabbit anti‐mouse Fn14, Nrf2, HIF‐1α, Ki67, or CD34 IgG (Table S2), were applied at 2 μg/mL, followed by incubation with polymer horseradish peroxidase‐conjugated goat anti‐rabbit IgG (DAKO Company, Glostrup, Denmark) at 2 mg/mL as the secondary antibody. The evaluation of epidermal thickness, cellular proliferation, and blood vessel density was performed as described previously [8, 9].

### 2.11. Superoxide Dismutase (SOD) Activity and Intracellular ROS Level

SOD activity was determined by using a ready‐made WST‐1 SOD Assay Kit (Dojindo, Kumamoto, Japan) [19]. WST‐1, a water‐soluble 2‐(4‐iodophenyl)‐3‐(4‐nitrophenyl)‐5‐(2,4‐disulfophenyl)‐2H‐tetrazolium salt, forms a formazan dye (450 nm absorbance) upon reaction with superoxide anions. The assay measures SOD/mimetics’ inhibition of WST‐1 reduction. Reactions at 37°C used serial dilutions to determine half maximal inhibitory concentration (IC_50_), from which SOD‐like activity was calculated (IC_50_ × initial dilution). One SOD unit (1 U) equals the enzyme amount in 20 μL, causing 50% inhibition. Inhibition rate and activity (U/mg) followed the manufacturer’s protocols.

Intracellular ROS levels were assessed using a 2^′^, 7^′^‐dichlorofluorescein diacetate (DCFH‐DA) method [19]. Cells in 6‐well plates underwent various pretreatments, followed by 1 mM hydrogen peroxide (Daxiong, Tianjin, China) stimulation for 1 h. This served as the positive control for detecting intracellular ROS levels. ROS production was then measured with a DCFH‐DA probe kit (Beyotime) per the manufacturer’s protocol. H_2_O_2_ levels in culture media were determined using a commercial H_2_O_2_ Assay Kit (Beyotime Biotechnology, Shanghai, China) before and after surface CAP treatment [14].

### 2.12. Statistical Analysis

Quantitative data are expressed as median with interquartile range or mean ± standard error of mean (SEM). Group comparisons were analyzed by one‐way analysis of variance (ANOVA) followed by Tukey’s post hoc tests, or Student’s *t*‐test, with statistical significance defined as *p*  < 0.05 (GraphPad Prism 8.01, GraphPad Software, San Diego, CA, USA).

## 3. Results

### 3.1. CAP Treatment Enhances Both Fn14 Expression and Cell Proliferation of HFSCs

**Figure 1:**
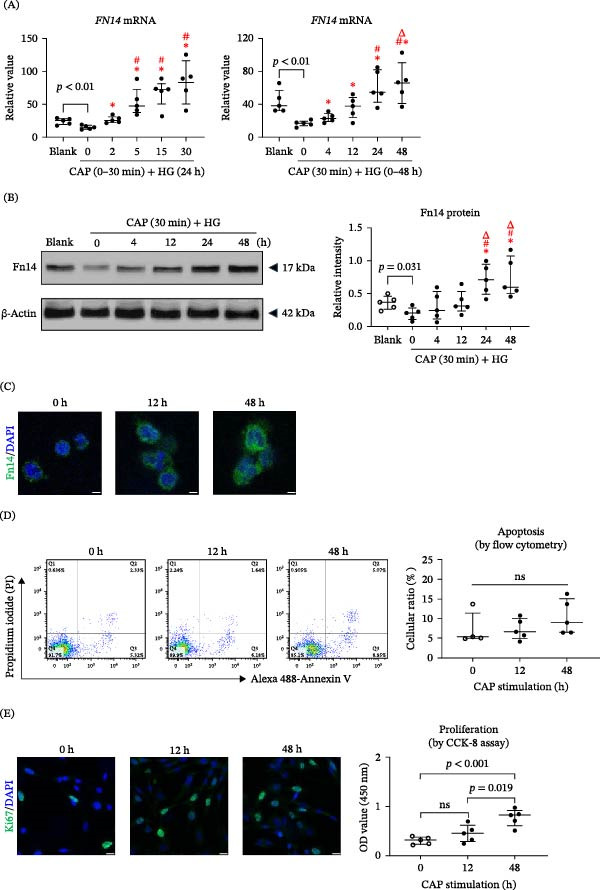
CAP treatment enhances both Fn14 expression and cell proliferation of HFSCs. HFSCs were cultured for 24 h in media that received 0–30 min CAP pretreatment. Some cells were cultured for 0–48 h in media that received 30‐min CAP treatment. (A) By qRT‐PCR, the mRNA expression level of *FN14* was determined in HFSCs. (B) By Western blotting, the protein expression level of Fn14 was detected in lysate of HFSCs, followed by ImageJ software quantitation. (C) The Fn14 expression pattern was analyzed by immunofluorescence. (D) The apoptotic ratios of HFSCs were measured by flow cytometry. (E) The proliferating cells were also detected by Ki67 staining, and quantitated by the CCK‐8 method. Scale bar = 10 μm. *n* = 5 per group. Error bars indicate median with interquartile range. Group comparisons were analyzed by one‐way ANOVA followed by Tukey’s post hoc tests.  ^∗^ *p* < 0.05, compared with 0 h group; ^#^ *p* < 0.01, compared with 2 (4) h group; Δ*p* < 0.001, compared with 5 (12) h group.

It is known that TWEAK/Fn14 interaction regulates the expression of differentiation, proliferation, and secretory functions of HFSCs [17]. We successfully isolated and identified human primary HFSCs, which were subsequently cultured in high‐glucose or CAP‐pretreated medium. It was found that high glucose microenvironment suppressed the mRNA expression of *FN14* while CAP pretreatment or treatment promoted *FN14* mRNA expression in time‐dependent manner (Figure 1A). By Western blotting, the expression of Fn14 protein increased in HFSCs in media pretreated with CAP for 30 min with culturing time (Figure 1B). This phenomenon was also reflected at 0, 12, and 48 h time points in immunofluorescent experiments (Figure 1C). We further explored the effect of CAP‐treated media on HFSCs at these three time points. It showed that CAP treatment exhibited no effect on cellular apoptosis while enhancing cellular proliferation (Figure S4; Figure 1D,E). These results suggested that CAP treatment positively regulates the Fn14 expression and cell proliferation of HFSCs.

### 3.2. CAP Treatment Upregulates Intracellular ROS and SOD Levels of HFSCs

**Figure 2:**
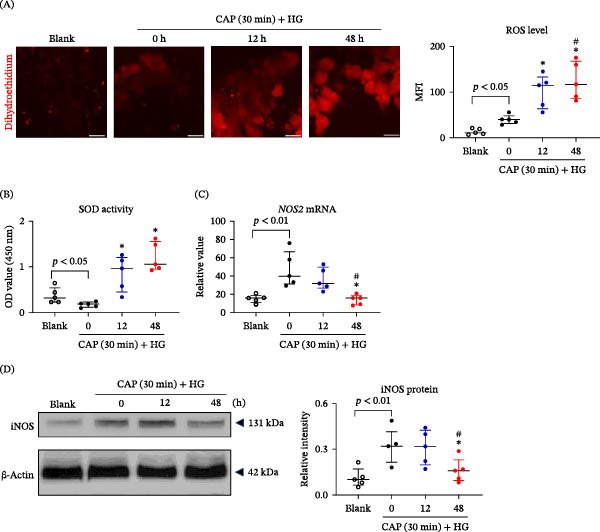
CAP treatment upregulates intracellular ROS and SOD levels of HFSCs. Human HFSCs were cultured in media that was treated with CAP. (A) By using DCFH‐DA probe, intracellular ROS levels were measured in HFSCs. (B) By using an SOD assay method, the SOD activity was analyzed in cells. (C) The mRNA expression level of *NOS2* (encoding iNOS) was determined by qRT‐PCR. (D) By Western blotting, the protein expression level of iNOS was detected in cell lysate, followed by ImageJ software quantitation. MFI, mean fluorescent intensity. Scale bar = 10 μm. *n* = 5 per group. Error bars indicate median with interquartile range. Group comparisons were analyzed by one‐way ANOVA followed by Tukey’s post hoc tests.  ^∗^ *p* < 0.05, compared with 0 h group; ^#^ *p* < 0.01, compared with 12 h group.

The delayed healing of diabetic wounds is associated with oxidative stress as well as mitochondrial dysfunction [22]. A recent report showed that CAP treatment favors the curing of diabetic wounds involving the regulation of the oxidative stress mediators iNOS [23]. In this study, we further investigated the effect of CAP on intracellular ROS and relevant molecules in HFSCs. It was found that the CAP‐treated media increased intracellular ROS level in a time‐dependent manner (Figure 2A). Moreover, it enhanced the activity of SOD in cells, which was preliminarily suppressed under high‐glucose (HG) microenvironment (Figure 2B). In accordance, the mRNA and protein expression levels of iNOS were elevated in HG media while they were decreased in CAP‐treated cells (Figure 2C,D). Therefore, CAP treatment increased total ROS level in HFSCs possibly due to exogenous ROS uptake since these cells exhibited higher SOD activity and lower iNOS levels accordingly.

### 3.3. CAP Treatment Modulates the Sirt1/Nrf2 Expressions and Activates the Wnt/β‐Catenin Signals in HFSCs

**Figure 3:**
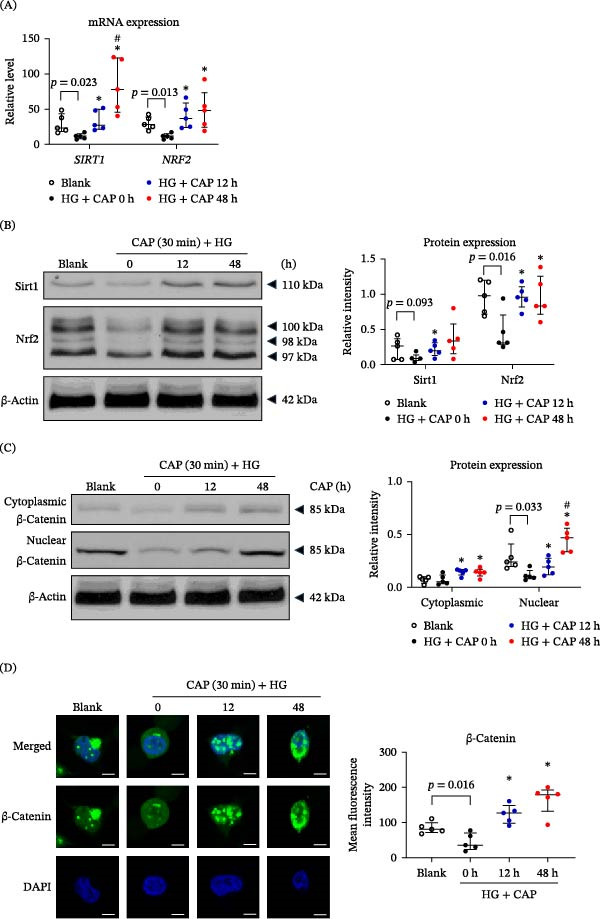
CAP treatment modulates the Sirt/Nrf2 expressions and activates the β‐catenin signals in HFSCs. HFSCs were cultured in media that was pretreated with CAP. (A) By qRT‐PCR analysis, the mRNA expression levels of *SIRT1*/*NFE2L2* (encoding Nrf2) molecules were determined in HFSCs. (B) By Western blotting, the protein expression levels of Sirt1 and Nrf2 were analyzed in cells. (C) The protein expression of β‐catenin was determined by Western blotting. (D) The localization of β‐catenin was visualized by immunofluorescence, and the fluorescence intensity was quantified using ImageJ software. (A) *n* = 5 per group. In (B, C), *n* = 3 per group. Scale bar = 5 μm. Error bars indicate median with interquartile range. Group comparisons were analyzed by one‐way ANOVA followed by Tukey’s post hoc tests.  ^∗^ *p* < 0.05, compared with 0 h group; ^#^ *p* < 0.01, compared with 12 h group.

It has been accepted that the nuclear factor Nrf2 suppresses oxidative stress in cells and itself is inhibited in the skin tissue of diabetic wounds [24]. Sirt1 promotes the dissociation of the Nrf2 protein from Keap1 and its subsequent translocation into the nucleus through deacetylation modification [25]. We analyzed the expressions of Sirt1 and Nrf2 in HFSCs stimulated with CAP. We observed that CAP treatment increased their mRNA and protein expression levels, which were inhibited exactly under the HG microenvironment (Figure 3A,B). Next, we explored the Wnt/β‐catenin signals that possibly mediate the regulation of Sirt/Nrf2 expressions. It was shown that HG reduced the nuclear expression of β‐catenin protein. However, CAP treatment amplified both cytoplasmic and nuclear expressions of β‐catenin, which was even more prominent in nuclear fractions (Figure 3C). Such enhancement effect of CAP treatment on the cytoplasmic expression and nuclear translocation of β‐catenin was also mirrored in immunofluorescent experiments (Figure 3D). Thus, CAP treatment upregulates Sirt1/Nrf2 expression and enhances Wnt/β‐catenin signaling in HFSCs.

### 3.4. Both Downstream Cytokines and Proliferation‐Relevant Proteins Are Affected by CAP Treatment

**Figure 4:**
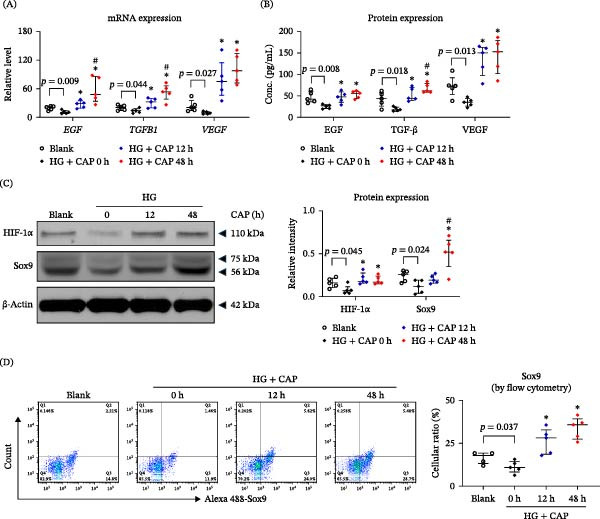
Both downstream cytokines and proliferation‐relevant proteins are affected by CAP treatment. HFSCs were cultured in media that was pretreated with CAP. (A) By qRT‐PCR analysis, the mRNA expression levels of certain cytokines and molecules were determined in cells. (B) By ELISA, the levels of several cytokines were measured in culture supernatants. (C) By Western blotting, the expression levels of HIF‐1α and Sox9 proteins were analyzed in cell lysates. (D) By flow cytometry, the expression level of Sox9 (Q2 and Q3 indicating positive cells) was analyzed in HFSCs. *n* = 5 per group. Error bars indicate median with interquartile range. Group comparisons were analyzed by one‐way ANOVA followed by Tukey’s post hoc tests.  ^∗^ *p* < 0.05, compared with 0 h group; ^#^ *p* < 0.01, compared with 12 h group.

Recently, we reported that TWEAK induces the expressions of EGF, TGF‐β, and VEGF in HFSCs [17]. In this study, we identified similar enhancement effect of CAP treatment on the secretion of these cytokines at both mRNA and protein levels (Figure 4A,B). The transcription regulator HIF‐1α promotes wound healing pathways while it is suppressed in wound tissue under HG microenvironment [26]. We further found that CAP treatment increased the protein expression level of HIF‐1α in cultured HFSCs (Figure 4C). As a downstream molecule of HIF‐1α activation [27], Sox9 protein exhibited similar alteration in response to CAP treatment (Figure S4; Figure 4C,D). These experiments also verified the inhibitory effect of HG on both the HIF‐1α and Sox9 expressions. Hence, CAP treatment reversed such HG’s suppression of the downstream cytokines and proliferation‐relevant proteins in HFSCs.

### 3.5. CAP‐Activated Hydrogel Promotes Tissular Fn14 Expression and Wound Healing in a Mouse Model

**Figure 5:**
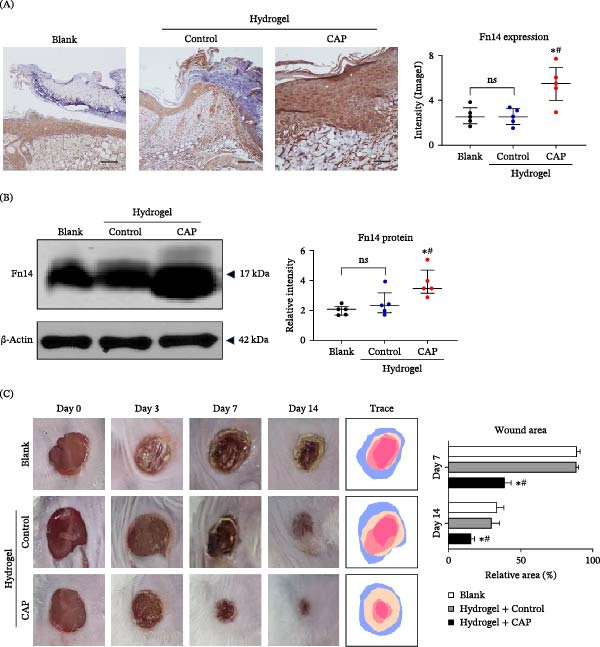
CAP‐activated hydrogel promotes tissular Fn14 expression and wound healing in a mouse model. The full‐layer incision wounds were created in wild‐type mice. (A) By immunohistochemistry, the expression of Fn14 was detected on day 7 in wound or perilesional tissue. Bar = 25 μm. (B) By Western blotting, the protein expression level of Fn14 was determined in wound tissue. (C) Representative images of wounds are shown at different time points. *n* = 5 per group. Scale bar = 25 μm. Error bars indicate median with interquartile range (A,B) or mean ± SEM (C). Group comparisons were analyzed by one‐way ANOVA followed by Tukey’s post hoc tests.  ^∗^ *p* < 0.05, compared with blank group; ^#^ *p* < 0.01, compared with control group.

To investigate the in vivo effect of CAP on skin tissue, we created full‐layer incision wounds in wild‐type diabetic mice. Some mice were treated with hydrogel that was pre‐activated with CAP. By immunohistochemistry, we observed that the expression of Fn14 was significantly enhanced on day 7 in the CAP‐activated hydrogel group as compared to the blank (no hydrogel treatment) or control (non‐activated hydrogel treatment) group (Figure 5A). Moreover, the epidermal thickness, cellular proliferation ratio (Ki67‐positive) and blood vessel density (CD34‐positive) were higher in the CAP‐activated hydrogel group (Figure S5). Such changes of Fn14 expression were also reflected by Western blotting analysis (Figure 5B). The healing of diabetic wounds was observed at different time points, showing that CAP‐activated hydrogel‐treated mice had less wound area on day 7 or 14 after the establishment of skin wounds (Figure 5C). There was no significant difference between the other two groups. Obviously, CAP‐activated hydrogel but not vehicle hydrogel promoted tissular Fn14 expression and wound healing in a type 1 diabetic mouse model.

### 3.6. Fn14 Signaling Mediates the Enhancement Effect of CAP Treatment on Wound Healing

**Figure 6:**
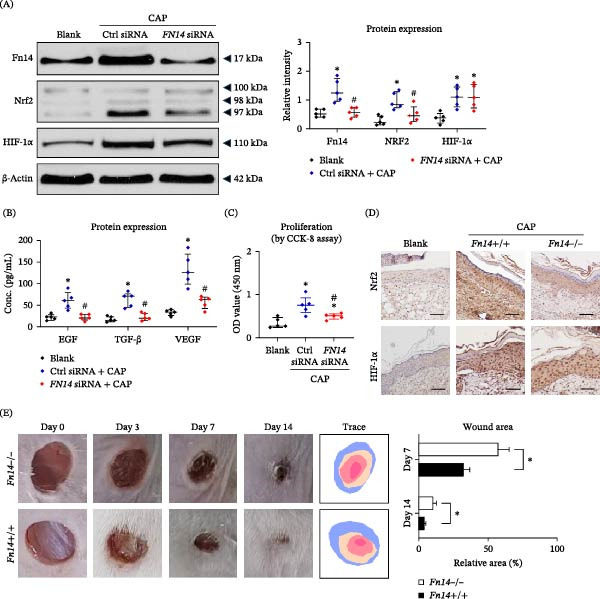
*Fn14* deficiency abrogates the enhancement effect of CAP‐activated hydrogel on cultured cells or wound healing in mice. HFSCs were cultured in HG medium that was pretreated with CAP. Some cells were pre‐transfected with control or *FN14* siRNA. (A) These cells were then analyzed for the protein expressions of Nrf2 and HIF‐1α. (B) The concentrations of certain cytokines were determined in culture supernatants. (C) Cellular proliferation ratios were analyzed by a CCK‐8 method. (D) The full‐layer incision wounds were created in wild‐type mice. The expressions of Nrf2 and HIF‐1α were detected on day 7 by immunohistochemistry. Bar = 25 μm. (E) The wound areas were measured at different time points. *n* = 5 per group. Scale bar = 25 μm. In (A–C), error bars indicate median with interquartile range. Group comparisons were analyzed by one‐way ANOVA followed by Tukey’s post hoc tests.  ^∗^ *p* < 0.05, compared with blank group; ^#^ *p* < 0.01, compared with control group. In (E), error bars indicate mean ± SEM. Group comparisons were analyzed by Student’s *t*‐test.  ^∗^ *p* < 0.05.

Since CAP treatment modulated the expressions and translocation of multiple proteins, we performed additional experiments to identify the key mediator underlying such modulation. By Western blotting analysis, *FN14* siRNA transfection abrogated the CAP enhancement of Nrf2 expression in cultured HFSCs. However, HIF‐1α was unaffected in these CAP‐treated cells despite *FN14* inhibition (Figure 6A). Moreover, the expression levels of downstream cytokines including EGF, TGF‐β, and VEGF were significantly promoted by CAP under control conditions, and markedly attenuated upon *FN14* inhibition (Figure 6B). Cellular proliferation ratio was partially mitigated in *FN14* siRNA‐transfected cells that received CAP treatment (Figure 6C). In the full‐skin wound model of wild‐type mice, the expressions of Nrf2 and HIF‐1α increased on day 7 with CAP treatment while declined in *Fn14*‐deficient strain (Figure 6D). Both epidermal thickness and blood vessel density were also higher in wild‐type mice (Figure S5). Consistently, these wound areas were smaller at the time points of days 7 and 14 (Figure 6E). These data indicated that the enhancement effect of CAP treatment on wound healing was at least partially mediated by Fn14 signaling.

## 4. Discussion

In this study, we demonstrated that CAP treatment enhances both Fn14 expression and cell proliferation in HFSCs. In vitro studies verified that CAP induces HFSC proliferation and cytokine production. At the molecular level, CAP modulates Sirt1/Nrf2 expression and activates the Wnt/β‐catenin signaling pathway in cells, subsequently affecting downstream cytokines and proliferation‐related proteins. Furthermore, in vivo experiments using CAP‐activated hydrogel confirmed its ability to promote tissular Fn14 expression and accelerate wound healing in a mouse model. Importantly, Fn14 signaling has been identified as the key mediator of CAP’s enhancing effects on HFSC activity and wound repair, establishing a mechanistic link between CAP treatment and regenerative outcomes.

Previous studies have shown that TWEAK/Fn14 activation regulates interfollicular epidermal stem cells and contributes to the healing of diabetic skin wounds [11, 28]. Also, inhibition of Fn14 signaling delays the repair of burn wounds accompanied by the suppression of growth factor production and extracellular matrix synthesis [9]. Topical administration of recombinant TWEAK accelerates healing of burn wounds in mice [29]. Recently, we found that TWEAK regulates the functions of HFSCs, involving the proliferation, migration, and cytokine production, via the Fn14‐Wnt/β‐catenin‐chemokine (C‐X‐C motif) 4 signaling axis [17]. It is consistent with prior reports of reduced Wnt/β‐catenin signaling activity in tissues from mouse models of type 1 or type 2 diabetes mellitus [30, 31]. In fact, Sirt1 promotes the dissociation of Nrf2 from Keap1 [25], and induces intracellular β‐catenin deacetylation [30], thereby activating Wnt/β‐catenin signaling. These results from current study were consistent with previous reports. Besides, we elucidated the regulatory effect of TWEAK on HFSCs under the HG condition mimicking diabetic status. Our findings demonstrated for the first time that diabetic microenvironment suppresses Fn14 signaling in HFSCs, suggesting a potential target for the management of diabetic wounds.

Although the therapeutic outcome of CAP treatment was observed preliminarily on patients with diabetic foot ulcers [12, 13], its precise mechanism underlying such CAP action remains unclear. Moreover, there was no report about the effect of CAP on HFSCs, which play a critical role in the skin wound healing process [4]. In this study, we found that CAP treatment enhances the expressions of Fn14 as well as the ROS generation regulators Nrf2 and HIF‐1α in wound tissue. In fact, Nrf2 signaling is suppressed by oxidative stress in mouse models of type 1 and type 2 diabetes mellitus [32, 33]. Diabetic wounds exhibit delayed healing process due to oxidative stress and ROS overgeneration [22]. Nrf2 suppresses oxidative stress in cells while it is less expressed in the skin tissue of diabetic wounds [24]. As a transcription regulator, HIF‐1α maintains redox homeostasis by suppressing intracellular ROS generation and activating antioxidant pathways [31]. HIF‐1α is also repressed in injured tissue of diabetic wounds [26]. However, our results showed that both Nrf2 and HIF‐1α are upregulated in either HG‐treated HFSCs or diabetic wounds upon CAP stimulation. Our findings are also supported by prior evidences that CAP exhibits protective effects against oxidative stress in mouse models of diabetes mellitus [15, 23]. Therefore, our findings proved that the CAP exerts therapeutic effect via recovering antioxidant activities in cells.

Different from previous reports that CAP interventions were conducted using direct plasma or plasma‐activated water [34], CAP‐activated hydrogel was applied to the wound surface in this study. In fact, such active hydrogel offers the advantages of simpler administration and greater patient acceptance compared with direct CAP application [14]. This gel preparation may even maintain a moist environment, enable controlled release of ROS or reactive nitrogen species ingredients, and prevent and control infections. We observed that CAP‐activated hydrogel but not vehicle hydrogel promoted tissular Fn14 expression and wound healing in a mouse type 1 diabetic model. Moreover, the enhancement effect of CAP treatment was largely negated in *Fn14*‐deficient mice. In accordance, the CAP’s upregulation of the Nrf2 and HIF‐1α expression and the secretion of EGF, TGF‐β, and VEGF was abrogated in cultured HFSCs. Obviously, Fn14 signaling mediates the regulative or therapeutic effect of CAP on HFSCs or diabetic wounds.

Mitochondrial ROS generated by oxidative stress trigger activation of the nuclear factor‐κB pathway, leading to sustained upregulation of iNOS expression and forming a positive feedback loop [32]. The expression of iNOS is upregulated in the diabetic wounds, which can be retained by CAP treatment [23]. Our results were consistent with those findings. We further observed that CAP stimulation initially amplifies and then suppresses the expression of iNOS in HFSCs under HG condition. The enhancement effect of HG on iNOS level and CAP‐induced initial upregulation followed by subsequent suppression of iNOS expression were also verified in other cell types or tissue [32, 33]. These findings indicated that CAP disrupts the positive feedback loop of ROS generation possibly through suppressing intracellular iNOS level.

**Figure 7:**
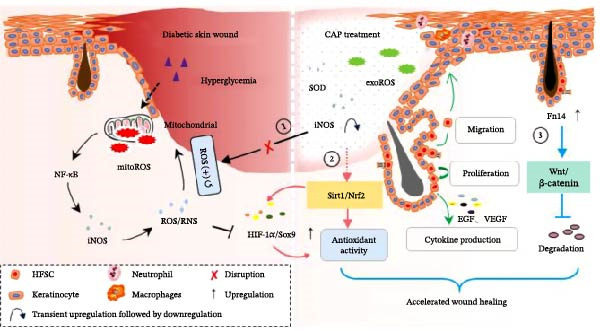
CAP regulation of cellular ROS signals in accelerating wound healing. In diabetic HG conditions, mitochondrial ROS activates nuclear factor kappa B (NF‐κB), driving sustained iNOS upregulation and a ROS/NF‐κB/iNOS positive feedback loop, while suppressing Nrf2 and HIF‐1α antioxidant activity. CAP‐activated hydrogel therapeutically: (1) disrupts this loop via exogenous ROS that induces a biphasic response—transient iNOS enhancement followed by persistent suppression; (2) restores antioxidant capacity by activating Sirt1/Nrf2 to elevate Nrf2 and HIF‐1α; and (3) triggers Fn14 signaling to activate Wnt/β‐catenin in HFSCs, stabilizing cytoplasmic β‐catenin. Together, these mechanisms enhance HFSC proliferation, migration, and EGF/VEGF secretion, accelerating diabetic wound healing.

Some discrepancies seem to exist between previous reports and our present findings. For example, TWEAK knockdown caused an enhancement of local Nrf2 expression level in an allergic conjunctivitis model [35]. Also, CAP preferentially reduced protein expression of HIF‐1α and blocked phosphorylation of mammalian target of rapamycin/Akt proteins in myeloid leukemia cells [36]. CAP treatment increased both tissular and serum levels of Nrf2 while decreased the tissular level of HIF‐1α in a vitiligo mouse model [34]. We speculate that TWEAK knockdown does not necessarily reduce Fn14 expression and may even induce its feedback upregulation. CAP can modulate cellular HIF‐α expression in a hydrogen peroxide dose‐dependent manner [14]. Moreover, the decline of HIF‐α expression in vitiligo tissue might result from the depletion of CD8^+^ T lymphocytes, a secondary phenomenon following CAP treatment [14]. Therefore, in this investigation, the observed upregulation of Fn14, HIF‐1α, and Nrf2 expression induced by CAP treatment is mechanistically plausible. These ROS‐related molecules are affected by CAP as shown in Figure 7.

This study had several limitations. Specifically, we did not investigate the impact of CAP on HFSCs in vivo. The employed cell and animal models could not fully replicate the inflammatory microenvironment of diabetes. Additionally, this mouse model fails to recapitulate some features of human type 1 diabetes mellitus, including immune‐mediated β‐cell destruction, progressive complications, and autoimmune markers. Also, its design omitted considering bacterial infection factors in skin wounds affecting the healing process. Future studies should focus on elucidating the effects of different CAP formulations or dosages on HFSCs and diabetic wounds, with plans to accelerate clinical application trials.

In conclusion, CAP enhances HFSC proliferation and Fn14 expression in vitro, inducing cytokine secretion. In parallel, CAP activates the Wnt/β‐catenin pathway and modulates the Sirt1/Nrf2 axis to regulate downstream effectors. In vivo, CAP‐activated hydrogel promotes tissue‐level Fn14 expression and accelerates diabetic cutaneous wound healing in a murine model. Fn14 signaling is the key mediator, mechanistically linking CAP to HFSC activation and tissue repair. This study provides a therapeutic blueprint for diabetic wound regeneration using CAP‐mediated approaches.

## Author Contributions

**Xiaoyan Zou:** conceptualization, investigation, methodology, formal analysis, writing – original draft. **Lingling Peng:** formal analysis, project administration. **Mai Luo:** investigation. **Ziqing Qu:** investigation. **Guanglei Hu:** investigation. **Xiaoming Liu:** resources, project administration, writing – review & editing. **Zhu Yan:** investigation. **Shruti Pokharel:** writing – review & editing. **Yumin Xia:** conceptualization, writing – review & editing. **Fangyan Jia:** conceptualization, project administration, writing – review & editing.

## Funding

Yumin Xia was supported by the National Natural Science Foundation of China (No. 82173445). Guanglei Hu was supported by the National Natural Science Foundation of China (No. 82404110) and the Basic Research Fund Project of Xi’an Jiaotong University (No. xzy012025123). Lingling Peng was supported by the Natural Science Foundation of Shaanxi Province (No. 2024JC‐YBQN‐0810). Fangyan Jia was supported by the Natural Science Foundation of Shaanxi Province (No. 2025JC‐YBMS‐1073). Xiaoming Liu was supported by the Shenzhen Science and Technology Program (No. JCYJ20220530114204010) and the Key Research Plan of Nanshan Health Bureau (No. NSZD2024061). Xiaoyan Zou was supported by the Research Grant of Maternal and Child Health Hospital of Hubei Province (No. 2023SFYZ003).

## Conflicts of Interest

The authors declare no conflicts of interest.

## Supporting Information

Additional supporting information can be found online in the Supporting Information section.

## Supporting information


**Supporting Information** Supporting Information are available at *Journal of Inflammation Research* online. Figure S1: Transfection efficiency of siRNA reagents. (A) The expression of Fn14 was detected by Western blotting, followed by ImageJ software quantitation. (B) The expression pattern of Fn14 was detected in HFSCs by immunofluorescence. Data are from three independent experiments. Representative images are shown. Scale bar = 5 μm. Error bars indicate median with interquartile range. Group comparisons were analyzed by one‐way ANOVA followed by Tukey’s post hoc tests. Figure S2: The preparation of CAP‐activated hydrogel and CAP stimulation of culture media. (A) The diagram of CAP generation and hydrogel or culture medium activation. (B) CAP preparation device used in this study, and on‐site photographs of gel activation process. (C) Activated hydrogel was topically applied to skin wounds once daily. Figure S3: The determination of ROS/RNS in CAP‐activated culture media. (A) The concentrations of H_2_O_2_ were determined in phenol red‐free media activated by CAP for 0 to 60 min. ns, not significant.  ^∗∗∗^ *p* < 0.001. (B) The concentrations of NO, NO₂⁻, and NO₃⁻ were determined in these media accordingly. No significant differences were observed between the 1‐ and 60‐min groups, although their concentrations were higher than that of the 0‐min group. Error bars indicate mean ± SEM. Group comparisons were analyzed by one‐way ANOVA followed by Tukey’s post hoc tests. Figure S4: Flow cytometry gating strategies for identifying HFSC subpopulations. (A) Identification of Annexin V/PI‐stained cells. (B) Identification of cells stained with Alexa Fluor 488‐conjugated anti‐SOX9 antibody. Figure S5: Histological evaluation of skin tissue from a full‐thickness excisional wound model. HE staining and immunohistochemical analysis, including epidermal proliferation (Ki67‐positive cells) and angiogenesis (CD34‐positive vessels), were performed in wounds tissue on day 7 post‐injury. Representative images show (A, B) comparative pathological changes between vehicle control and CAP‐activated hydrogel treatments on wild‐type mice, and (C, D) detailed morphological features under CAP‐activated hydrogel treatment in both wild‐type and Fn14‐deficient mice. ns, not significant. Scale bar = 25 μm. *n* = 5 per group.  ^∗^ *p* < 0.05;  ^∗∗^ *p* < 0.01;  ^∗∗∗^ *p* < 0.001. Error bars indicate median with interquartile range. Group comparisons were analyzed by one‐way ANOVA followed by Tukey’s post hoc tests. Table S1: Primers used for qRT‐PCR analysis. Table S2: Antibodies used for immunohistochemistry, Western blotting or immunofluorescent assay.

## Data Availability

All data are presented in the figures and supporting file. There are no data available in a public, open access repository.
