# Supplementary material for: Cold Atmospheric Plasma Enhances Fn14 Signaling in Hair Follicle Stem Cells, Thereby Promoting the Healing of Diabetic Skin Wounds in a Mouse Model
Source: J Immunol Res. 2026 May 5;2026:9082774. doi: 10.1155/jimr/9082774 (PMC13139759; doi:10.1155/jimr/9082774)
Supplement: Supplementary file 1 — Supporting Information Supporting Information are available at Journal of Inflammation Research online. Figure S1: Transfection efficiency of siRNA reagents. (A) The expression of Fn14 was detected by Western blotting, followed by ImageJ software quantitation. (B) The expression pattern of Fn14 was detected in HFSCs by immunofluorescence. Data are from three independent experiments. Representative images are shown. Scale bar = 5 μm. Error bars indicate median with interquartile range. Group comparisons were analyzed by one‐way ANOVA followed by Tukey’s post hoc tests. Figure S2: The preparation of CAP‐activated hydrogel and CAP stimulation of culture media. (A) The diagram of CAP generation and hydrogel or culture medium activation. (B) CAP preparation device used in this study, and on‐site photographs of gel activation process. (C) Activated hydrogel was topically applied to skin wounds once daily. Figure S3: The determination of ROS/RNS in CAP‐activated culture media. (A) The concentrations of H2O2 were determined in phenol red‐free media activated by CAP for 0 to 60 min. ns, not significant. ∗∗∗ p < 0.001. (B) The concentrations of NO, NO₂⁻, and NO₃⁻ were determined in these media accordingly. No significant differences were observed between the 1‐ and 60‐min groups, although their concentrations were higher than that of the 0‐min group. Error bars indicate mean ± SEM. Group comparisons were analyzed by one‐way ANOVA followed by Tukey’s post hoc tests. Figure S4: Flow cytometry gating strategies for identifying HFSC subpopulations. (A) Identification of Annexin V/PI‐stained cells. (B) Identification of cells stained with Alexa Fluor 488‐conjugated anti‐SOX9 antibody. Figure S5: Histological evaluation of skin tissue from a full‐thickness excisional wound model. HE staining and immunohistochemical analysis, including epidermal proliferation (Ki67‐positive cells) and angiogenesis (CD34‐positive vessels), were performed in wounds tissue on day 7 post‐injury. Represe [file JIMR-2026-9082774-s001.pdf]

Supplementary file

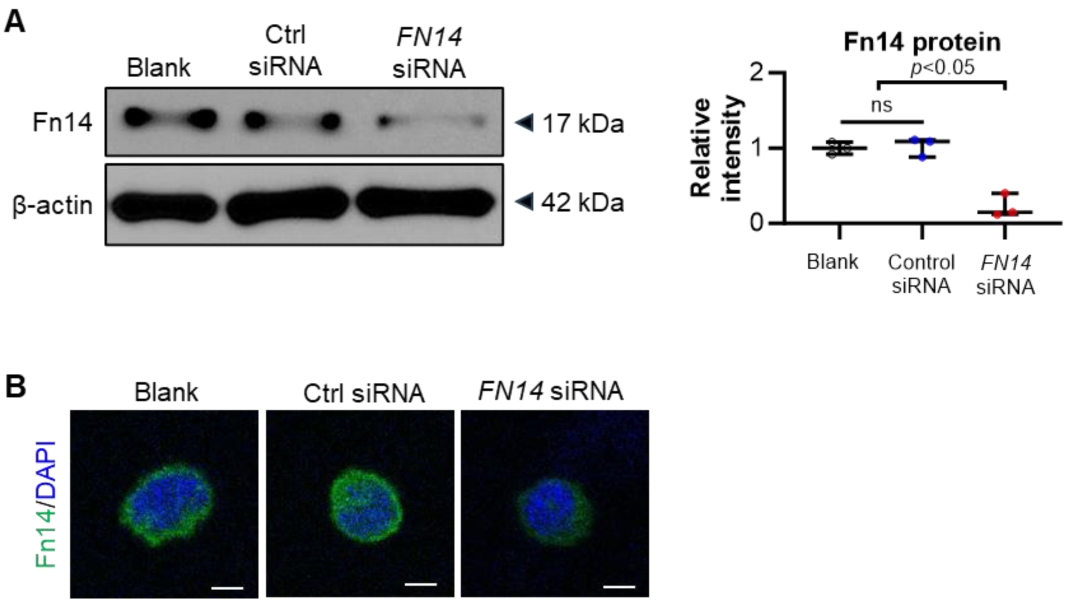

Supplementary Figure S1. Transfection efficiency of siRNA reagents. (**A**) The expression of Fn14 was detected by Western blotting, followed by ImageJ software quantitation. (**B**) The expression pattern of Fn14 was detected in HFSCs by immunofluorescence. Data are from three independent experiments. Representative images are shown. Scale bar = 5 µm. Error bars indicate median with interquartile range. Group comparisons were analyzed by one-way ANOVA followed by Tukey's post hoc tests.

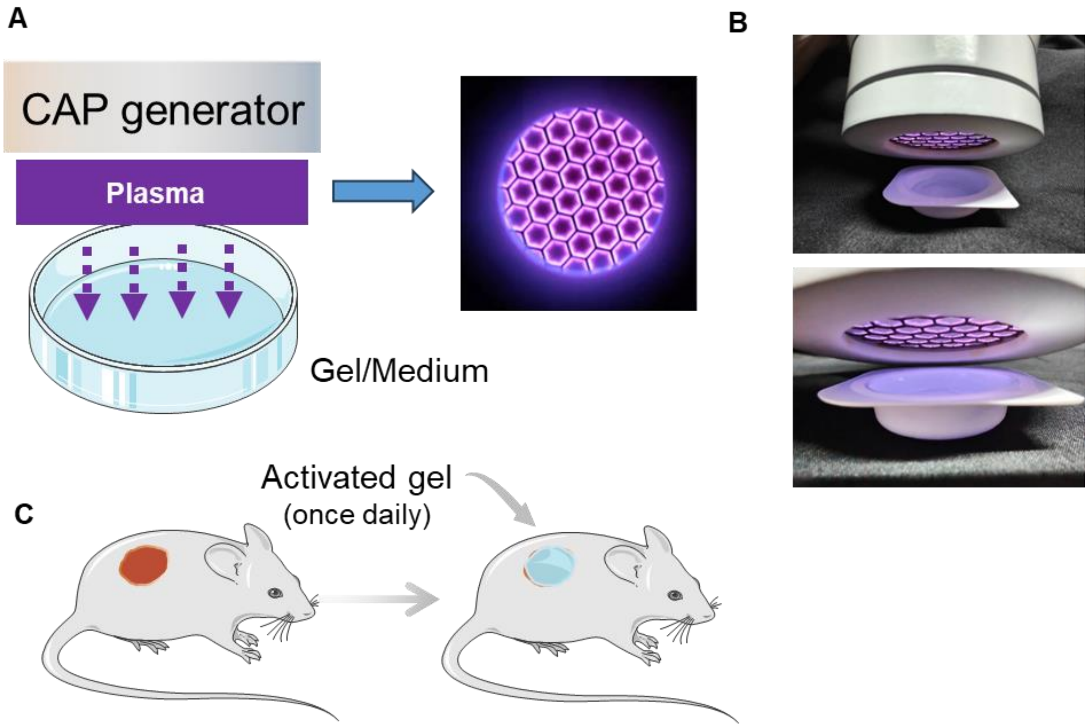

Supplementary Figure S2. The preparation of CAP-activated hydrogel and CAP stimulation of culture media. (**A**) The diagram of CAP generation and hydrogel or culture medium activation. (**B**) CAP preparation device used in this study, and on-site photographs of gel activation process. (**C**) Activated hydrogel was topically applied to skin wounds once daily.

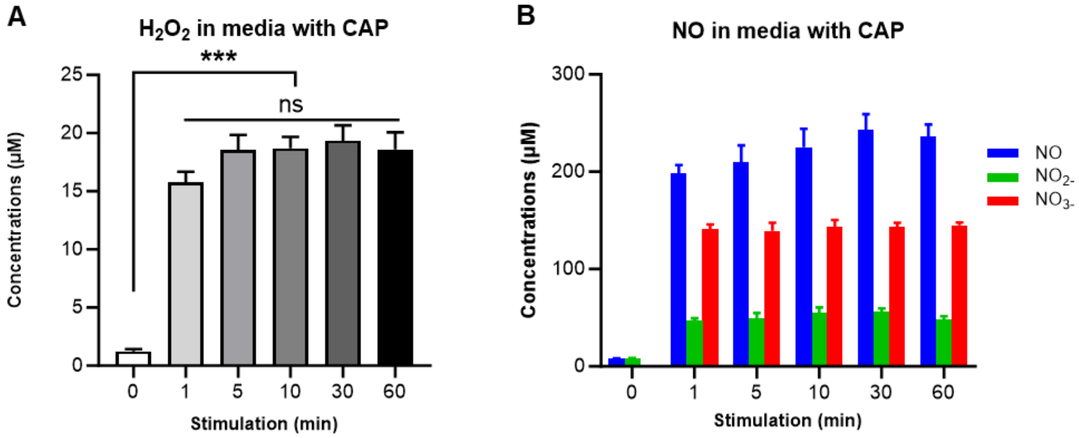

Supplementary Figure S3. The determination of ROS/RNS in CAP-activated culture media. (**A**) The concentrations of H_2O_2 were determined in phenol red-free media activated by CAP for 0 to 60 min. ns, not significant. ***p < 0.001. (**B**) The concentrations of NO, NO_2^-, and NO_3^- were determined in these media accordingly. No significant differences were observed between the 1- to 60-min groups, although their concentrations were higher than that of the 0-min group. Error bars indicate mean ± SEM. Group comparisons were analyzed by one-way ANOVA followed by Tukey's post hoc tests.

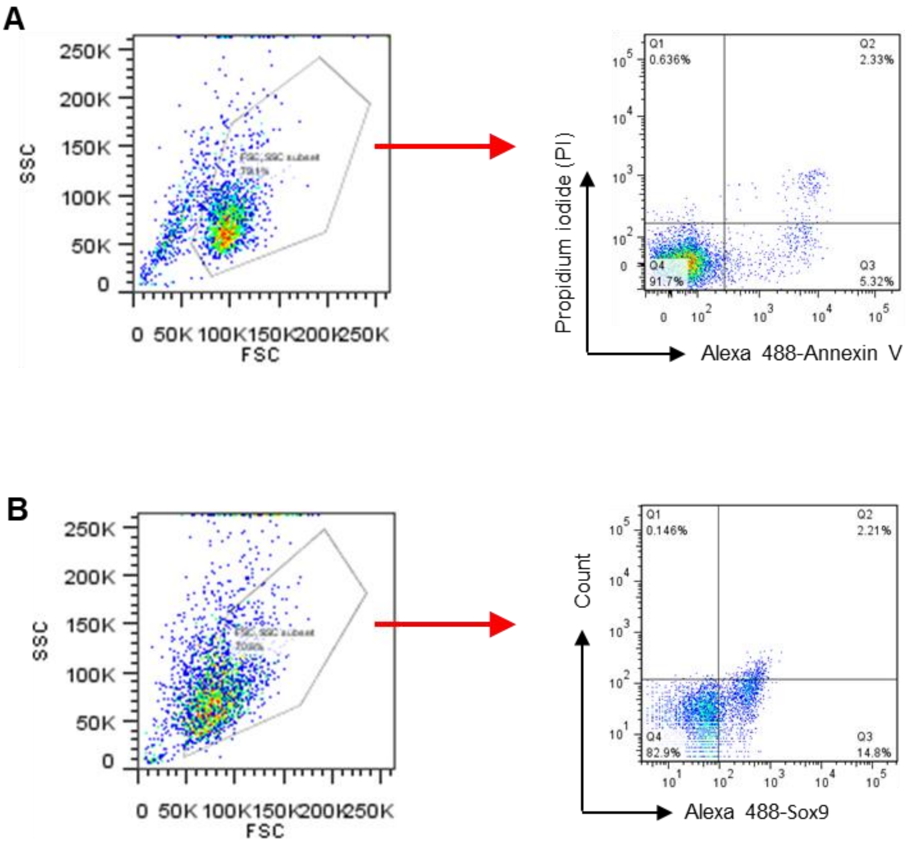

Supplementary Figure S4. Flow cytometry gating strategies for identifying HFSC subpopulations. (**A**) Identification of Annexin V/PI-stained cells. (**B**) Identification of cells stained with Alexa Fluor® 488-conjugated anti-SOX9 antibody.

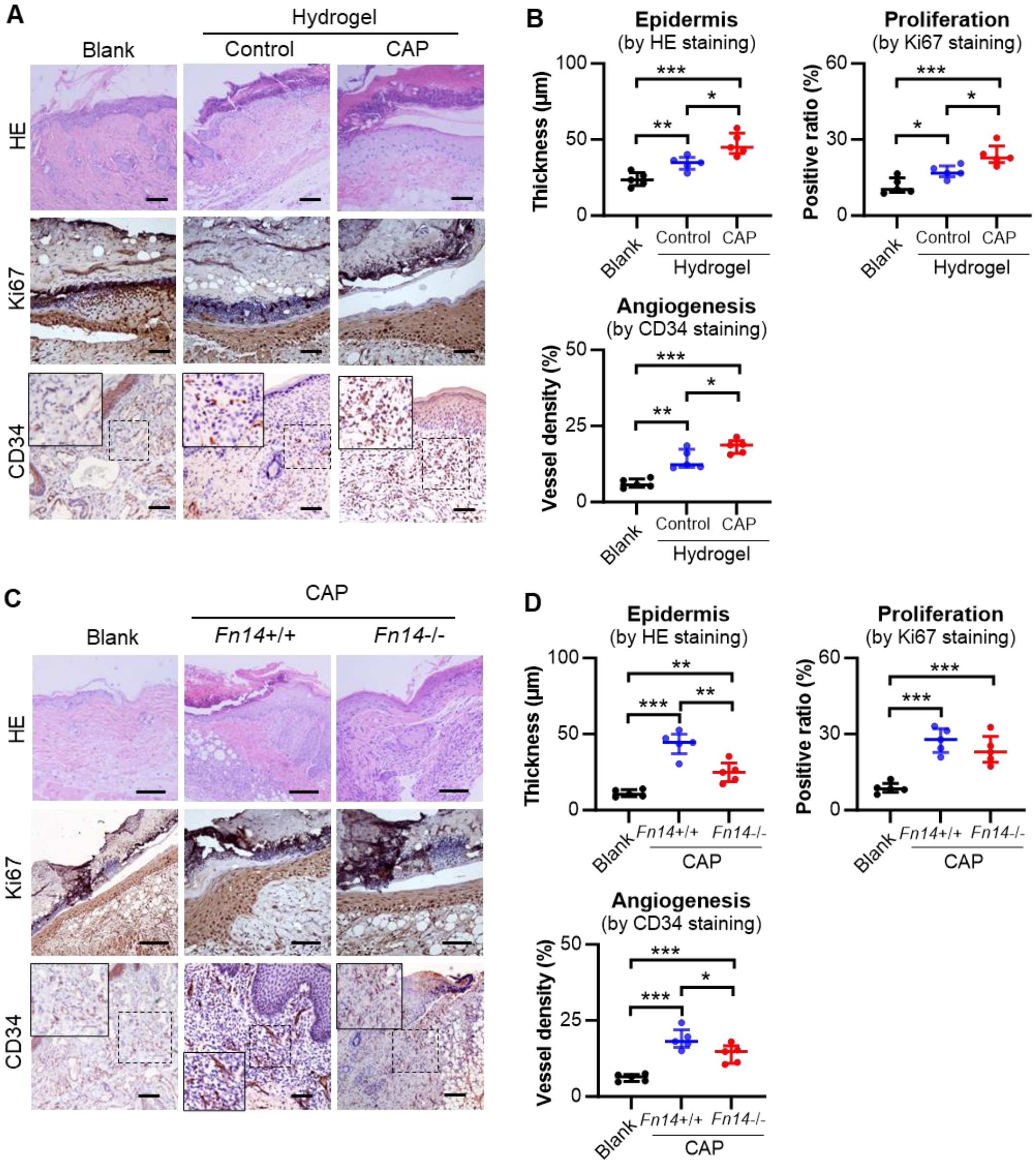

Supplementary Figure S5. Histological evaluation of skin tissue from a full-thickness excisional wound model. HE staining and immunohistochemical analysis, including epidermal proliferation (Ki67-positive cells) and angiogenesis (CD34-positive vessels), were performed in wounds tissue on day 7 post-injury. Representative images show (**A**, **B**) comparative pathological changes between vehicle control and CAP-activated hydrogel treatments on wild-type mice, and (**C**, **D**) detailed morphological features under CAP-activated hydrogel treatment in both wild-type and *Fn14*-deficient mice. ns, not significant. Scale bar = 25 μm. n = 5 per group. *$p < 0.05$; **$p < 0.01$; ***$p < 0.001$. Error bars indicate median with interquartile range. Group comparisons were analyzed by one-way ANOVA followed by Tukey's post hoc tests.

Supplementary Table S1. Primers used for qRT-PCR analysis

Gene	Species	Forward (5' to 3')	Reverse (5' to 3')
EGF	Human	AGAGGGAGAGGATGCCACAT	ACAAACCAAGGTTGAGGGCA
FN14	Human	CTCTGAGCCTGACCTTCGTG	GGGGGCACATTGTCACTGGA
GAPDH	Human	GCACCGTCAAGGCTGAGAAC	TGGTGAAGACGCCAGTGGA
NFE2L2	Human	CACGGTCCACAGCTCATCAT	GGTTGGGGTCTTCTGTGGAG
NOS2	Human	TCCAAGGTATCCTGGAGCGA	CAGGGACGGGAACTCCTCTA
SIRT1	Human	AGTTGGAAGATGGCGGACG	TTCGAGGATCTGTGCCAATCA
TGFB1	Human	CGGATCTCTTCCTGCTCGAC	CACAGTAGTAGGCGGCGTAG
VEGF	Human	AGGCCAGCACATAGGAGAGA	ACGCGAGTCTGTGTTTTTGC

Note: *NFE2L2* codes Nrf2 protein; *NOS2* codes iNOS protein; *TGFB1* codes TGF-β protein.

Supplementary Table S2. Antibodies used for immunohistochemistry, Western blotting or immunofluorescent assay

Antibody	Company	Catalog number	Application
β-actin	Abcam	ab124964	WB
β-catenin	Abcam	ab305261	WB
β-catenin	Abcam	ab194118	IF
CD34	Abcam	ab81289	IHC
iNOS	Abcam	ab178945	WB
Ki67	Abcam	ab281847	IF
Ki67	Abcam	ab16667	IHC
Fn14	CST	4403	WB, IHC
Fn14	Santa Cruz	sc-56250	IF
Mouse IgG	Abcam	ab150125	IF
Sirt1	CST	9475	WB
Nrf2	Abcam	ab313825	WB, IHC
HIF-1α	CST	48085	WB, IHC
Sox9	CST	82630	WB
Sox9	Abcam	ab196450	IF

Note: The Fn14 antibody (Santa Cruz, #sc-56250) was conjugated to Alexa Fluor® 488 by using a Lightning-Link® kit (Abcam, #ab236553) before its application for immunofluorescence.

Abbreviations: IF, immunofluorescence; IHC, immunohistochemistry; WB, Western blotting; CST, Cell Signaling Technology (company)
